# Anastomosing hemangioma: report of two renal cases and analysis of the literature

**DOI:** 10.1186/s13000-017-0597-4

**Published:** 2017-01-24

**Authors:** Marina Perdiki, Galateia Datseri, George Liapis, Nikolaos Chondros, Ioannis Anastasiou, Maria Tzardi, Johanna K. Delladetsima, Elias Drakos

**Affiliations:** 1 First Department of Pathology, Medical School, National and Kapodistrian University of Athens, Goudi, Mikras Asias 75, 11527 Athens, Greece; 2 Department of Pathology, Medical School, University of Crete, Voutes, Heraklion, 71110 Greece; 3 Department of Urology-University Hospital of Heraklion, Medical School, University of Crete, Voutes, Heraklion, 71110 Greece; 4 First Department of Urology, Medical School, National and Kapodistrian University of Athens, Goudi, Mikras Asias 75, 11527 Athens, Greece

**Keywords:** Anastomosing, Hemangioma, Renal, Non-renal, Review

## Abstract

**Background:**

Anastomosing hemangioma (AH) is a very rare vascular tumor mimicking angiosarcoma, predominately observed in kidney and less frequently in other organs. We present two new renal cases of AH at opposite ends of the clinical presentation spectrum, provide review of the literature and compare the epidemiological, clinical and pathological profiles of renal and non-renal cases.

**Case presentation:**

The first occurred in a 64-year-old woman presented with back pain and the second, a multifocal lesion, in a 47-year-old man with end stage renal disease (ESRD). Histology disclosed a vascular tumor with striking anastomosing pattern, minimal nuclear atypia and locally infiltrative pattern, mimicking superficially angiosarcoma. Extramedullary hematopoiesis, extensive perirenal fat entrapment and increased number of mast cells were additional features in the second lesion. Both patients are well, without disease, 25 and 14 months after diagnosis.

**Conclusion:**

Comprehensive review and analysis of the published literature show that the growing number of non-renal AHs exhibits similar epidemiologic, clinical, biologic and histologic characteristics with renal AHs and most mild differences vanish after exclusion of cases associated with ESRD. Better understanding of AH pathogenesis will contribute to optimal treatment choices.

## Background

In 2009, Montgomery and Epstein described 6 peculiar vascular lesions, 4 in the kidney or perirenal tissue and 2 in the testis, anatomical sites which rarely harbor angiomatous tumors, with histologic features mimicking angiosarcoma and evidence of benign behavior [1]. Because they consisted of capillary-size vessels with prominent anastomosing pattern, the term “anastomosing hemangioma (AH) of the genitourinary tract” was proposed. Since then, more than 50 renal cases, more frequently encountered in the context of end-stage renal disease (ESRD), including bilateral and multifocal lesions and approximately a similar number of extra-renal cases involving various internal organs, bone and soft tissue have been reported, mostly as individual cases or in small series [2–21].

Most lesions are accidental findings, frequently discovered during imaging studies for other tumors in the same, or other organs. All lesions are characterized histologically by anastomosing sinusoidal-like spaces, lined by a single file of CD31/CD34+ and D2-40- endothelial cells, some with hobnail morphology, supported by pericytes, accompanied by mild chronic inflammatory cells, without plasma cells and frequently associated with features of extra-medullary hematopoiesis [1–21]. They are also negative for the presence of human herpes virus 8 (HHV8). Occasionally, periodic acid–Schiff, diastase-resistant-positive (PAS-D+) hyaline globules, a secondary cavernous component, or increased number of mast cells have been reported [1, 14, 18].

In the present study, two renal cases of AH will be reported in association with a comparative analysis of literature data including renal and extra-renal lesions.

## Case presentation

### Case 1

Computed tomography scan in a 64-year-old female presented with back pain, normal renal function and unremarkable clinical history revealed an 1 cm-diameter solid lesion in the upper pole of the right kidney, which was removed by partial nephrectomy. Histology revealed a vascular tumor. Recent follow-up information showed the patient is well, without evidence of disease, 25 months after initial diagnosis.

### Case 2

Computed tomography scan for monitoring abdominal aortic aneurysmal disease in a 47-year-old male, in dialysis for the last 5 months, because of ESRD due to systemic lupus erythematous (SLE), revealed a solid 2.5 cm-diameter lesion near the hilum of the left kidney. Histology, after total left nephrectomy, revealed a vascular tumor. In addition, numerous smaller lesions with similar histologic features were disclosed in the renal parenchyma and perirenal soft tissue. The patient is well, without evidence of disease, 14 months after diagnosis.

### Materials and methods

Formalin-fixed, paraffin-embedded tissue sections (4 μm in thickness) were stained with haematoxylin–eosin, Giemsa, PAS, PAS-D and Gomori’s reticulin stain. Following heat-induced epitope retrieval, slides were incubated with antibodies specific for: CD3, CD4, CD8, CD20, CD31, CD34, CD138, EMA, Melan-A, HMB-45, CAM5.2, SMA, HHF-35, D2-40, c-KIT, HHV8 and Ki67 (MIB1) (Dako, Glostrup, Denmark). Detection of the immunoreaction was performed using the EnVisionFlex kit and 3,3-diaminobenzidine/H_2_O_2_ (both from Dako).

Extended cross-reference search of PubMed database was performed. For comparison of continuous variables (age, tumor size) in renal and non-renal lesions the nonparametric Mann-Whitney test was used. For comparison of non-continuous variables (presenting symptoms and histologic features) the Fischer’s exact test was used. *P* < .05 was considered significant. Only significant correlations are reported. All calculations were carried out using the StatView software (Abacus Concepts, Inc, Berkeley, CA).

### Results

#### Case 1

**Fig. 1:**
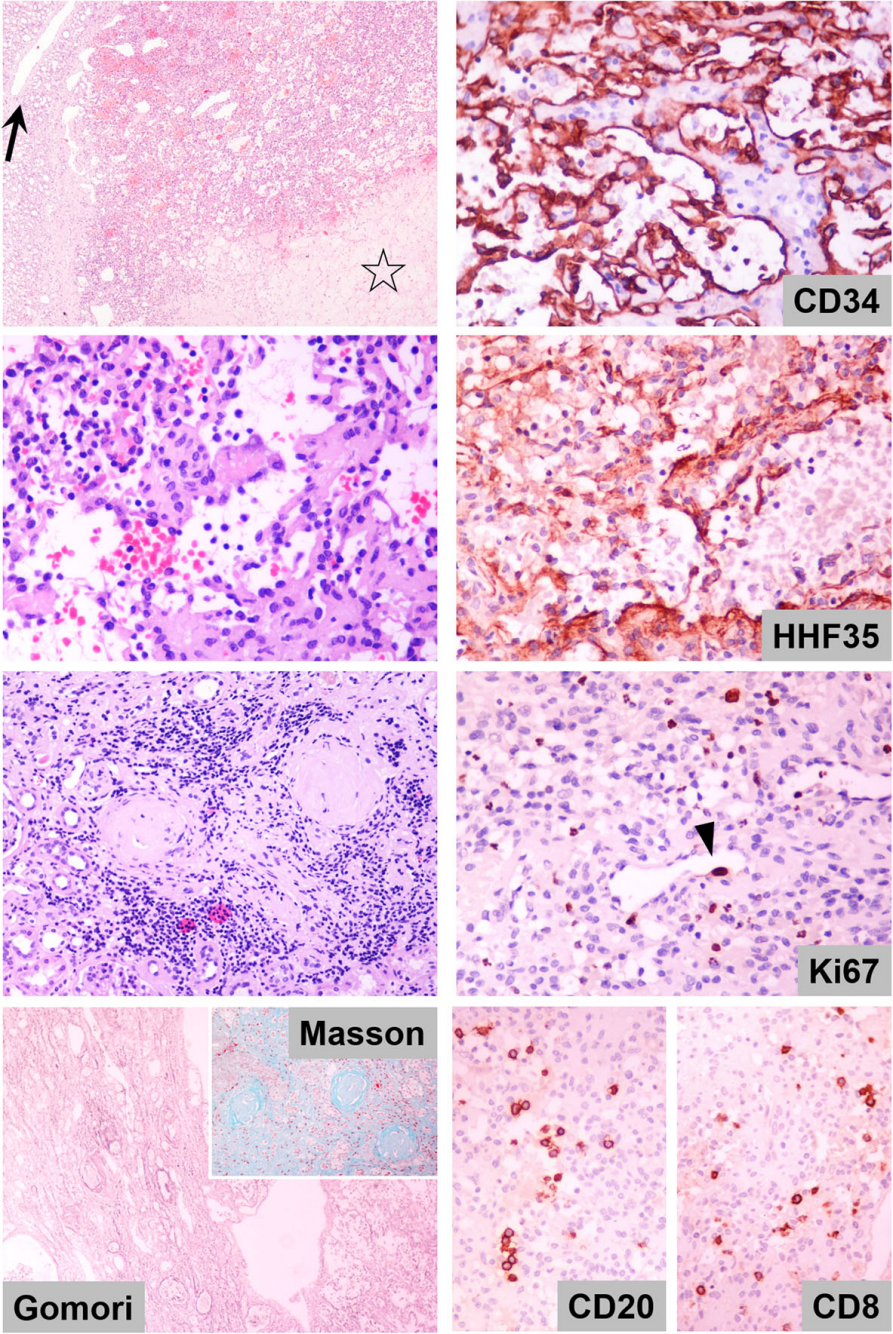
Histologic and immunohistochemical characteristics of case 1. (*Top left*): the architecture of the lesion was diffuse with areas of prominent sclerosis (asterisk). Large vessels (arrow) were observed at the periphery. (*Upper middle left*): the lesion was formed by small irregular angiomatous spaces with anastomosing pattern, lined by a simple layer of endothelial cells, some with hobnail morphology. (*Lower middle left*): prominent chronic inflammation accompanied by inter-tubular fibrosis and glomerular sclerosis was observed at the lesion margins (highlighted by histochemical Masson’s trichrome and Gomori’s reticulin staining in lower left). (*Top right*): endothelial cells were CD34-positive and bordered by HHF-35-positive pericytes (*upper middle right*). (*Lower middle right*): proliferation index (Ki67) was low, around 2–3% highlighting endothelial cells with enlarged nuclei (arrowhead). (Bottom right): Increased number of CD20+ B and CD8+ T lymphocytes (as well as CD4+ T-cells-not shown), but not plasma cells were observed all over the lesion. (3,3′ diaminobenzidine as chromogen, hematoxylin as counterstain, original magnifications 40X, 100X, 400X)

Grossly, the tumor, 1.1 cm in diameter, was solid, brownish, well demarcated, extending from the renal capsule at the cortex to the medulla and, focally, the surgical margins. Microscopically, it had diffuse architecture, with areas of fibrosis and myxoid degeneration of the stroma. It was characterized by capillary-size angiomatous spaces with prominent anastomosing pattern, lined by a single layer of endothelial cells with hobnail morphology, focally enlarged nuclei with dense chromatin and no mitosis, associated with foci of thrombosis and increased number of lymphocytes (Fig. 1). Lesion borders were well defined with increased number of larger vessels but without capsule. However, focally infiltrative pattern with entrapment of renal tubules, fibrosis of interstitial tissue, glomeruli sclerosis and increased number of lymphocytes were observed at the lesion margins. Renal parenchyma, distant to the lesion was unremarkable. Extramedullary hematopoiesis, hyaline globules, plasma cells, increased number of mast cells, or intravascular extension were not observed. Immunohistochemically, endothelial cells were CD31+, CD34+, D2-40-, CD8-, HHV8-, lined by SMA+ pericytes. Proliferation index, Ki67, was low, around 2–3%.

#### Case 2

**Fig. 2:**
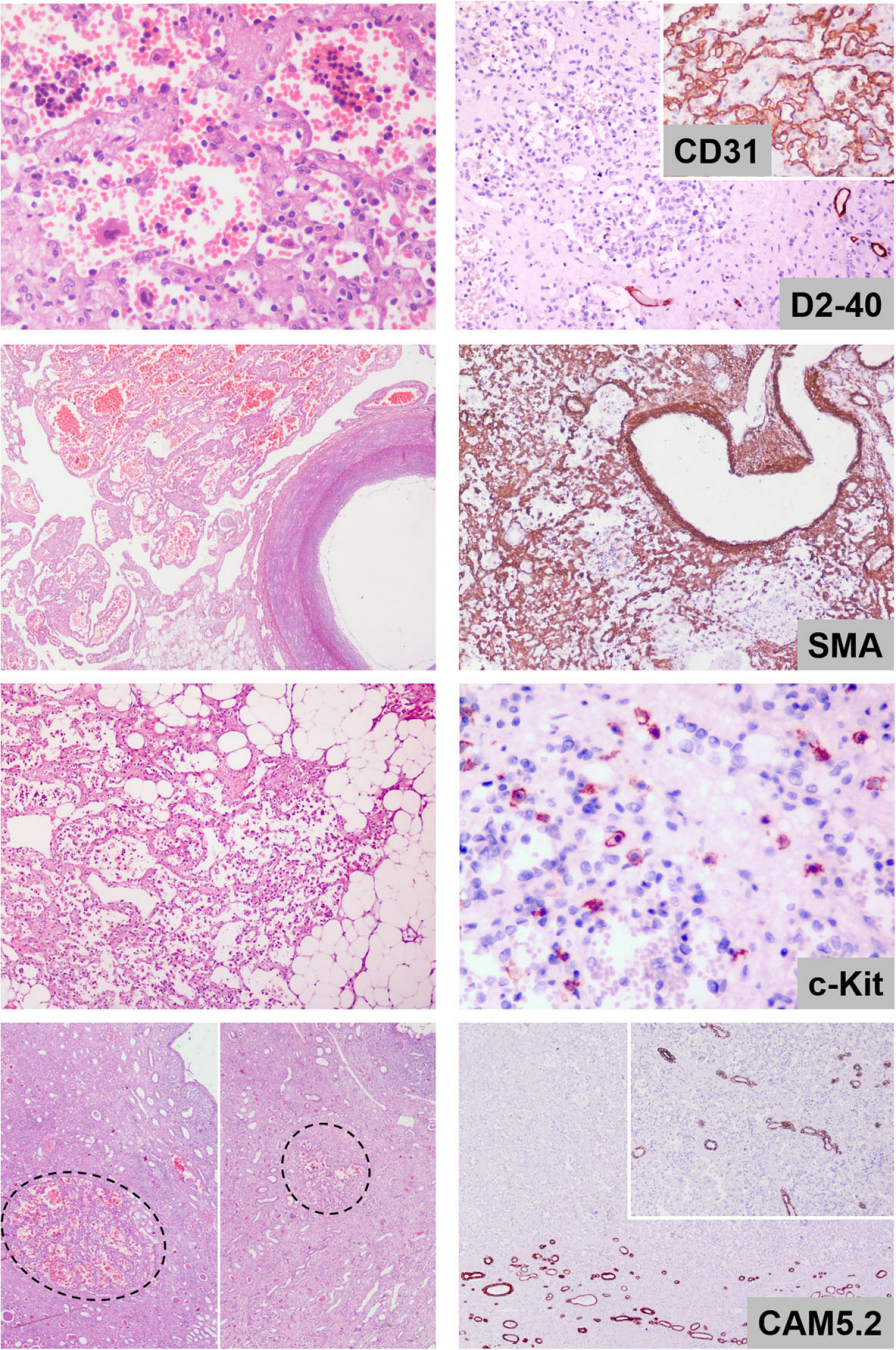
Histologic and immunohistochemical characteristics of case 2. (*Top left*): prominent hematopoietic islands were observed inside vascular spaces with typical anastomosing pattern. (*Upper middle left*): larger angiomatous spaces with less prominent anastomosing pattern were observed at the periphery of the largest lesion simulating cavernous hemangioma next to a large vessel. (*Lower middle left*): some lesions were extending to the sinus or peri-renal fat tissue entrapping adipocytes. (*Lower left*): numerous smaller lesions (circles) with similar histologic features were observed throughout renal parenchyma. (*Top right*): endothelial cells were D2-40-negative and CD31-positive (inset), while intervascular septa were highlighted by SMA-positive cells (*upper middle right*). (*Lower middle right*): c-kit staining highlighted increased number of mast cells all over the lesion. (*Bottom right*): Although at low power magnification the margins of the lesion seemed non-infiltrative, CAM5.2 immunostaining revealed locally infiltrative features with entrapment of renal tubules (inset) (3,3′ diaminobenzidine as chromogen, hematoxylin as counterstain, original magnifications 40X, 100X, 400X)

Grossly, the largest lesion, 2.8 cm in diameter, extending from the cortex to the medulla and the fat of the sinus was well demarcated. At least 15 similar lesions, 0.1–1.5 cm in diameter, throughout the parenchyma were observed. All lesions had histopathologic and immunophenotypic characteristics similar to case 1. That is, they consisted of small irregular angiomatous spaces with anastomosing and focally infiltrative pattern, lined by a single layer of CD31/CD34+ and D2-40- endothelial cells, some with hobnail morphology. Mild chronic lymphocytic inflammation and areas of fibrosis, or myxoid changes of the stroma were also detected. Hyaline globules, plasma cells, or intravascular extension were not observed. At the periphery of the largest lesion, larger angiomatous spaces with a less prominent anastomosing pattern simulating cavernous hemangioma were focally noticed. In addition, prominent extramedullary hematopoiesis, increased number of mast cells and entrapment of adipocytes were observed (Fig. 2). The rest of renal parenchyma showed features of ESRD with multiple epithelial cysts.

### Review and analysis of the literature

#### Epidemiologic profile

**Fig. 3:**
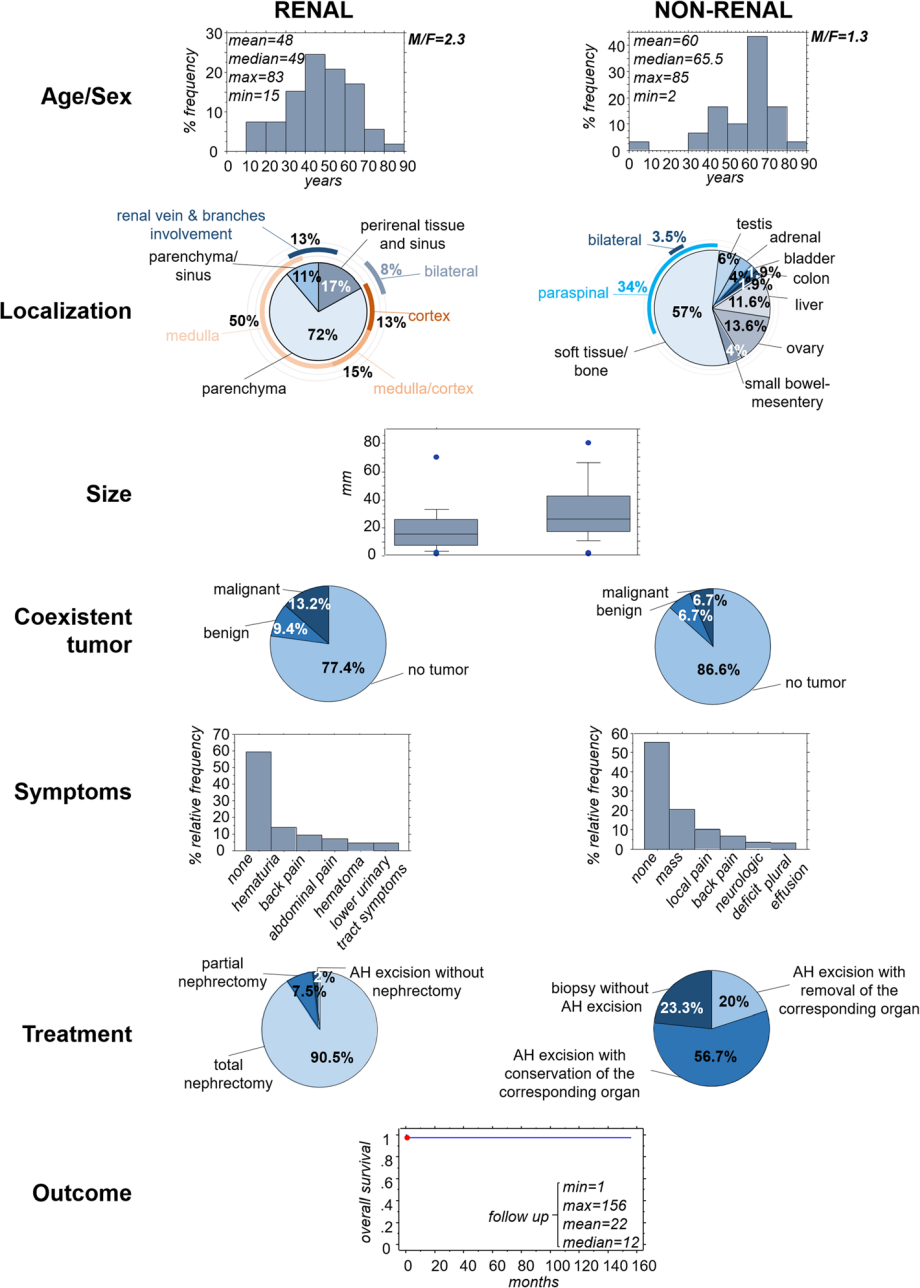
Epidemiologic, clinical, macroscopic, treatment and follow-up features of anastomosing hemangiomas published in the English literature. All information is derived from cases included in Table 1

Sixty-four renal (including the present cases) and 51 non-renal lesions with features of AH have been reported (Table 1). They occured in a wide range of ages from 2 to 85 years, with median age 49 years for renal and 65.5 years for non-renal cases (Mann-Whitney test, *p* = 0001). This difference vanishes if we exclude cases associated with ESRD (*p* = 0.3). The male to female ratio was 2.3 and 1.3 for renal and non-renal AH cases, respectively (Fig. 3).

**Table 1:** Anastomosing hemangiomas in the English literature

Renal		Non-renal		
Number^b^	Year/References	Site	Number^b^	Year/References
1–4	2009/ [1]	*Testis-spermatic cord*	1–3	2009/ [1, 21]^a^
5–9	2010/ [2]	*Ovary*	4–10	2011/ [3, 21]^a^
10–14	2011/ [3]	*Adrenal*	11–12	2012/ [17, 21]^a^
15–17	2012/ [4]	*Liver*	13–18	2013/ [18, 21]^a^
18	2012/ [5]	*Colon*	19	2013/ [18]
19	2012/ [6]	*Duodenum-mesentery*	20–21	2013/[18, 21]^a^
20–27	2013/ [7]	*Bladder*	22	2016/ [19]
28	2013/ [8]	*Soft Tissue*	23–51	2016/ [20, 21]^a^
29	2013/ [9]			
30	2014/ [10]			
31	2014/ [11]			
32	2014/ [12]			
33–34	2014/ [13]			
35–49	2014/ [14]^c^			
50	2015/ [15]			
51	2015/ [16]			
52–62	2016/ [21]^a^			
63–64	present			

^a^The 32 renal and non-renal cases presented in this recent study, except for localization and imaging features, are not accompanied by other clinical or pathologic characteristics and, therefore, except for localization, they are not included in the following analysis; ^b^multifocal lesions in the same kidney are numbered as one tumor, while bilateral lesions as two; ^c^some of the tumors in reference [14] are, also, described in references [1] and [2]

#### Sites of involvement and size

Most renal lesions (72%) were confined to renal parenchyma, more frequently localized to the medulla (50%) and less frequently to the medulla and cortex (15%), or the cortex (13%). A minority of cases was localized to the renal sinus (13%) or peri-renal tissue (17%), or involving renal vein branches (13%). Bilateral cases (8%) have been reported. Bone and soft tissue, mainly with para-spinal localization (34%) were the most frequent involved sites of non-renal AHs (57%). Sites with descending frequency included the ovary (14%), liver (12%), testis (6%), adrenal (4%), small bowel/mesentery (4%), colon (2%) and bladder (2%). Also, a single bilateral soft tissue case has been reported. The size of AHs varied from 1 mm to 8 cm (median size 1.5 cm for renal AH and 2.6 cm for non-renal AH, Mann-Whitney test *p* = 0.002).

#### Clinical context

A different tumor, malignant or benign, was found at the organ involved by AH at 23% and 13% of renal and non-renal cases, respectively, sometimes being the reason for the discovery of AH.

Almost 60% of renal and non-renal lesions were asymptomatic. Clinical manifestations with decreasing frequency included hematuria, back, or abdominal pain, hematoma and lower urinary tract symptoms for renal AHs and mass, local, or back pain, neurologic deficit and plural effusion for non-renal AHs.

**Fig. 4:**
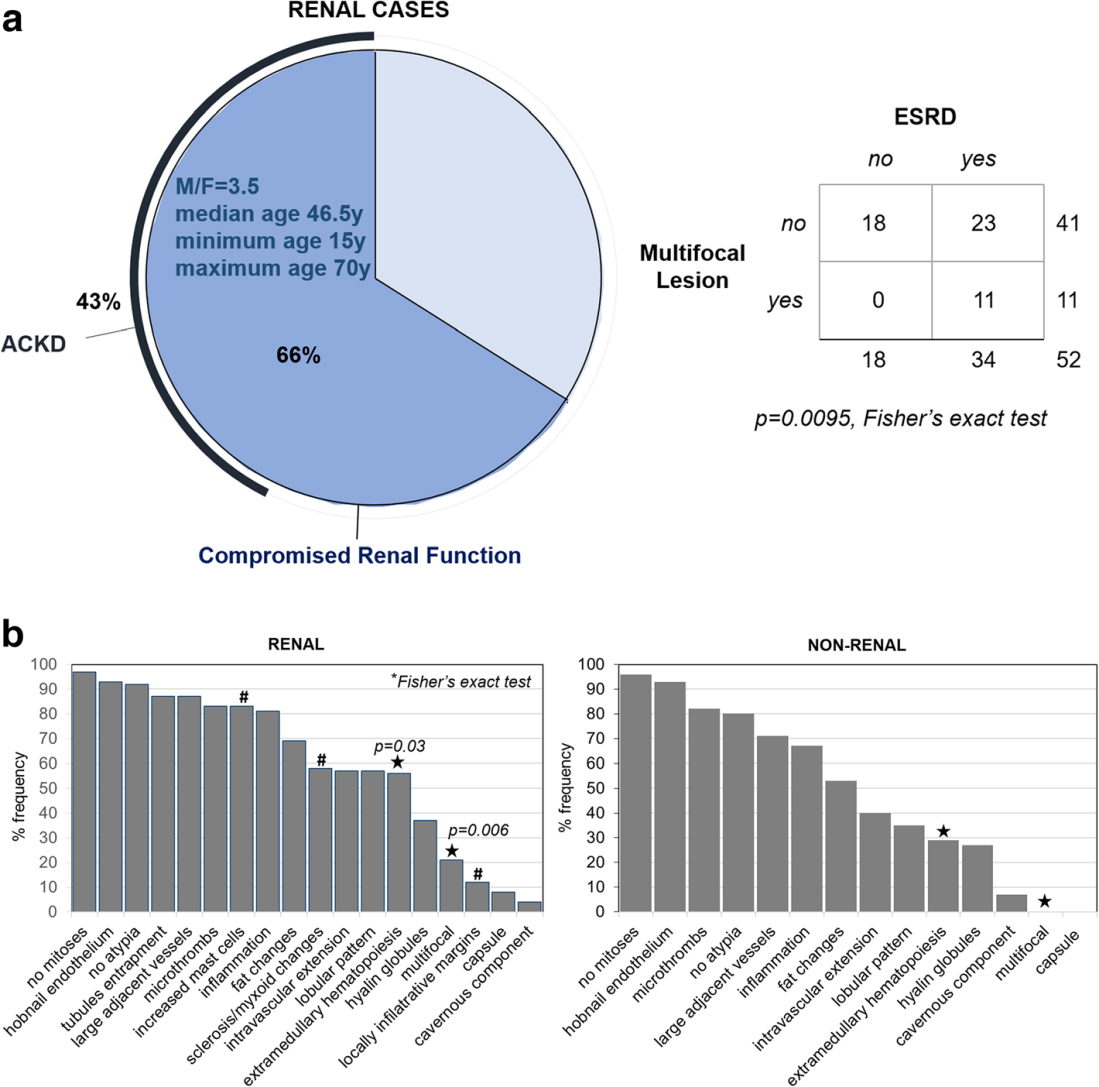
Histopathologic features of anastomosing hemangiomas published in the English literature. **a** Characteristics associated with end-stage renal disease. Almost two thirds of renal anastomosing hemangiomas have been reported in patients with compromised renal function, most in end stage renal disease (ESRD; on the *left*). Almost two thirds of these cases were accompanied by acquired cystic kidney disease (ACKD). All multifocal lesions have been reported exclusively in patients with compromised renal function (Fischer exact test *p* = 0.0095, on the right). **b** Comparison between renal and extra-renal lesions. Renal and extra-renal anastomosing hemangiomas show similar histologic features. # indicates characteristics not studied, or not reported in extra-renal lesions. Asterisk indicates characteristics statistically significantly more frequently observed in renal lesions (Fischer exact test *p* = 0.03 and *p* = 0.006)

Two thirds (66%) of renal AHs involved kidneys of patients with compromised renal function. In 43% of renal AHs, acquired cystic kidney disease (ACKD) was observed (Fig. 4a). Most of these patients were in dialysis and the causes of chronic renal failure varied widely including systemic and idiopathic renal diseases. All multifocal AHs were encountered in kidneys associated with ESRD (Fisher’s exact test, *p* = 0.0095).

#### Treatment and outcome

Most renal AHs have been treated with total nephrectomy (90%), while a more conservative treatment was more frequently used for non-renal AHs, with 23% of the patients left without additional treatment, following diagnostic biopsy.

Only one death was reported, one month after total nephrectomy, for reasons unrelated to the tumor. In all other cases with available follow-up information (median follow-up 12 months, minimum 1 month, maximum 156 months) no recurrence or death were reported (Fig. 3).

#### Histopathology

Histologic features of renal and non-renal AHs were very similar (Fig. 4b). Extra-medullary hematopoiesis was observed more frequently in renal lesions (Fisher’s exact test, *p* = 0.03). This association vanishes if we exclude ESRD-associated cases (Fisher’s exact test, *p* > 0.9-extramedullary hematopoiesis was observed in 27% of renal AHs without renal dysfunction and in 56% of ESRD-associated AH lesions, Fischer’s exact test, *p* = 0.01). Also, some features including stroma sclerosis or increased number of mast cells have not been reported, or studied in non-renal lesions.

The immunophenotype of the endothelial cells was identical in all lesions: CD31+, CD34+, Factor VIII+, FLI1+, GLUT1-, D2-40-, CD8- and HHV8-.

## Discussion

Well differentiated angiosarcoma is probably the most important lesion that must be successfully discriminated against anastomosing hemangioma, a task more demanding in limited biopsy material [1]. It is the irregular architectural pattern and the focally infiltrative features, including fat entrapment and intravascular extension of anastomosing hemangioma that may raise the possibility of an aggressive lesion. However, the lack of significant endothelial nuclear atypia and pleomorphism, lack or rarity of mitosis, low proliferative index, absence of necrosis or broadly infiltrative pattern, as well as the regular pericytic accompanying layer and the absence of multilayering endothelial formations are important features for correct diagnosis. Extramedullary hematopoiesis, a frequent finding in anastomosing hemangioma is an additional clue [1, 21].

There are, also, histologic features of anastomosing hemangioma shared by other vascular tumors, which, therefore, may be included in the differential diagnosis. Hyaline globules, PAS-D-positive structures, believed to be secondary lysosomes, observed in 37% of renal AHs and 27% of non-renal AHs, may be encountered in other types of benign vascular tumors, Kaposi sarcoma and angiosarcoma [22]. However, in contrast to Kaposi sarcoma, all anastomosing hemangiomas are negative for HHV-8 and lack increased number of plasma cells and the characteristic slit-like pattern [1–21]. Hobnailing of endothelial cells is, also, a feature of retiform hemangioendothelioma, a locally aggressive, but low grade vascular lesion and papillary intralymphatic angioendothelioma, known also as Dabska tumor, a rarely metastasizing neoplasm [22]. However, the first is characterized by a monomorphic arborizing vascular pattern and involves usually the skin and the subcutaneous tissue and the second shows characteristic intraluminal papillary tufts and positivity for the lymphatic endothelial marker D2-40, features not reported in anastomosing hemangioma [1–21]. The characteristic anastomosing pattern, reminiscent of spleen vasculature must be distinguished from splenosis in deep seated lesions. However, the lack of organized lymphoid tissue and negativity for CD8 assist easily in the discrimination of these two possibilities. Also, since renal lesions can be adipocyte-rich and entrap renal parenchyma, vascular-rich lesions of the kidney, including angiomyolipoma and renal cell carcinoma may be included in the differential diagnosis, a task easily accomplished with the use of immunohistochemical markers including HMB45, Melan-A, cytokeratins and CD10.

It must be added that, while anastomosing hemangioma is emerging as a frequent type of renal vascular lesion, different types of angiomatous lesions including common capillary and cavernous hemangioma, papillary intravascular endothelial hyperplasia, arteriovenous malformations and angiosarcoma may, also, be observed in the kidney [2, 4, 7, 14, 22].

Although kidney is the single organ, harboring more than half of all anastomosing hemangiomas reported so far, recent literature shows that this type of vascular lesion is increasingly observed in various parenchymal organs, with soft tissue and bone cases representing the most frequent extra-renal site of involvement [1, 17–21]. Although the number of the cases published is still relatively small for drawing definite conclusions, our review of the literature suggests that both renal and non-renal tumors show similar general epidemiological, clinical, macroscopic and histologic characteristics, (Fig. 3). Minor differences, like the younger average age in renal compared to non-renal cases may be explained by the frequent discovery of the lesion in surveys of end stage disease kidneys. Also, the more frequent association of renal cases with extramedullary hematopoiesis probably has to do with the frequent report of AH lesions in ESRD, since this association vanishes if we exclude ESRD-associated lesion. The more frequent presence of extramedullary hematopoiesis in ESRD-associated AH as compared with the rest of renal or exrarenal AHs may be due to pathogenetic factors of the preexisting renal disease, independent of AH development.

Anastomosing hemangioma pathogenesis remains unknown. Possible genetic alterations have not been studied. It is uncertain whether it is a clonal lesion, a true neoplasm, or a polyclonal, hyperplastic, deregulated vascular tissue. Also, the possible pathogenic role of ESRD, the more frequent associated clinical condition, remains obscure. It is highly improbable the occurrence of multifocal lesions exclusively in this context to be accidental. It is possible that additional pathogenetic factors contribute to the development of ESRD-associated AH. Whether, alternatively, a common factor between non-ESRD-associated and ESRD-associated cases, exaggerated in the last group contributes to the pathogenesis of anastomosing hemangioma remains to be studied.

Literature analysis provides strong evidence of the benign behavior of AH. This suggests that less aggressive treatment may be preferable. However, the minimalistic strategy (diagnostic biopsy and no treatment) should be weighed against the potential of significant tissue damage because of the growing lesion in the course of time [20, 21]. More informative imaging analysis may contribute to better decisions.

## Conclusion

In conclusion, anastomosing hemangioma seems to be a distinctive type of vascular tumor with similar clinical, biologic and histologic characteristics in renal and non-renal involvement sites. Growing knowledge and better understanding of its pathology may contribute to more accurate diagnosis and optimal treatment choices.

## Abbreviations

AH: Anastomosing hemangioma
ESRD: End stage renal disease
HHV8: Human herpes virus 8
PAS-D+: Periodic acid–Schiff, diastase-resistant-positive
SLE: Systemic lupus erythematous
